# Aflatoxin levels in sunflower seeds and cakes collected from micro- and small-scale sunflower oil processors in Tanzania

**DOI:** 10.1371/journal.pone.0175801

**Published:** 2017-04-18

**Authors:** Juma A. Mmongoyo, Felicia Wu, John E. Linz, Muraleedharan G. Nair, Jovin K. Mugula, Robert J. Tempelman, Gale M. Strasburg

**Affiliations:** 1 Department of Food Science and Human Nutrition, Michigan State University, East Lansing, Michigan, United States of America; 2 Department of Food Technology, Nutrition and Consumer Sciences, Sokoine University of Agriculture, Morogoro, Tanzania; 3 Department of Horticulture, Michigan State University, East Lansing, Michigan, United States of America; 4 Department of Animal Science, Michigan State University, East Lansing, Michigan, United States of America; Utah State University, UNITED STATES

## Abstract

Aflatoxin, a mycotoxin found commonly in maize and peanuts worldwide, is associated with liver cancer, acute toxicosis, and growth impairment in humans and animals. In Tanzania, sunflower seeds are a source of snacks, cooking oil, and animal feed. These seeds are a potential source of aflatoxin contamination. However, reports on aflatoxin contamination in sunflower seeds and cakes are scarce. The objective of the current study was to determine total aflatoxin concentrations in sunflower seeds and cakes from small-scale oil processors across Tanzania. Samples of sunflower seeds (n = 90) and cakes (n = 92) were collected across two years, and analyzed for total aflatoxin concentrations using a direct competitive enzyme-linked immunosorbent assay (ELISA). For seed samples collected June-August 2014, the highest aflatoxin concentrations were from Dodoma (1.7–280.6 ng/g), Singida (1.4–261.8 ng/g), and Babati-Manyara (1.8–162.0 ng/g). The highest concentrations for cakes were from Mbeya (2.8–97.7 ng/g), Dodoma (1.9–88.2 ng/g), and Singida (2.0–34.3 ng/g). For seed samples collected August-October 2015, the highest concentrations were from Morogoro (2.8–662.7 ng/g), Singida (1.6–217.6 ng/g) and Mbeya (1.4–174.2 ng/g). The highest concentrations for cakes were from Morogoro (2.7–536.0 ng/g), Dodoma (1.4–598.4 ng/g) and Singida (3.2–52.8 ng/g). In summary, humans and animals are potentially at high risk of exposure to aflatoxins through sunflower seeds and cakes from micro-scale millers in Tanzania; and location influences risk.

## Introduction

Aflatoxins are secondary metabolites produced by the fungi *Aspergillus flavus* and *Aspergillus parasiticus*, which commonly infect food crops such as maize, peanuts, and tree nuts. They cause liver cancer and aflatoxicosis in humans and animals. The fungi produce four main types of aflatoxin: aflatoxin B_1_ [AFB_1_], B_2_ [AFB_2_], G_1_[AFG_1_], and G_2_ [AFG_2_]. AFB_1_, the most carcinogenic mycotoxin, is typically produced in higher quantities than its counterparts. The International Agency for Research on Cancer (IARC) has classified “naturally occurring mixes of aflatoxins” as a Group 1 human carcinogen: known to cause cancer in humans [1].

Chronic exposure to aflatoxin contributes to increased incidence of liver cancer cases worldwide. It has been estimated that 25,000–155,000 humans die each year of liver cancer associated with chronic exposure to aflatoxins, through consumption of contaminated maize and peanuts [2]. Furthermore, chronic exposure to dietary aflatoxin is associated with immuno-suppression, stunted growth in children, and acute aflatoxicosis at high doses [3–5]. In the past, human and animal exposure to dietary aflatoxins in Sub-Saharan Africa was considered to be mainly through consumption of maize and groundnuts. However, consumption of oilseeds such as sunflower, sesame, and cotton may also contribute significantly to the overall human and animal exposure to aflatoxins through food and feed [6–8].

In Tanzania, sunflower is an oilseed crop that provides animal feed and cooking oil. The Central Sunflower Corridor (CSC), comprised of Mbeya, Iringa, Morogoro, Dodoma, Singida, Manyara and Karatu-Arusha leads in sunflower farming and sunflower micro-scale oil milling activities. A report by the Tanzanian Ministry of Agriculture, Food Security and Cooperative (MAFSC) indicates that annual production in 2008 was approximately 350,000 metric tons [9]. In 2015, production had increased about tenfold from 2008, due to an increased sunflower seed market. Therefore, the sunflower industry is an important contribution to the economics of Tanzania.

Small-scale sunflower farmers make a living by selling sunflower seeds to processors, who extract cooking oil and produce seed cakes, which consist of the seed residue following extraction of oil. In 2015, a 70-kg bag of sunflower seeds sold for 60,000 Tanzania Shillings (Tshs) (US$30) from which one can produce approximately 45 kg of cakes and 20 liters of crude oil for sale. A 5-kg loss might be due to the poor efficiency of the milling machines. Whereas humans eat roasted and salted seeds as a snack food, the cakes are used as animal feed for chickens, dairy cows, and goats. Dodoma, Singida, Arusha and Manyara are the major sunflower cake-producing regions in Tanzania; producing approximately 100,000 metric tons of sunflower cakes per year, which serve as a reliable source of animal feed for livestock in Northern Tanzania and Kenya.

Aflatoxin in animal feedstuffs has been a growing concern in the dairy industry due to the prevalence of aflatoxin M_1_ (hydroxylated form of AFB_1_) in dairy products from animals consuming AFB_1_-contaminated feed. Researchers in Kenya found 72% of 439 cow-milk samples they collected from urban Kenya contaminated with aflatoxin M_1_ [8]. Researchers in Tanzania also found aflatoxin M_1_ contamination in fresh cow milk retailed in Dar es Salaam [10] and in Singida [11]. Although aflatoxin M_1_ is less carcinogenic than aflatoxin B_1_, it also has demonstrated toxicological effects [12], and infants weaned on contaminated milk may be at high risk of exposure to aflatoxin M_1_ and its associated carcinogenic actions. Hence, the United States Food and Drug Administration (USFDA) has set an action level for aflatoxin M_1_ in milk of 0.5 ng/g, while its action level for total aflatoxins (aflatoxin B_1_ + B_2_ + G_1_ + G_2_) is 20 ng/g for human food and dairy-animal feed [13]. Dairy cows fed with feedstuffs and nursing humans fed with food contaminated with total aflatoxin levels higher than 20 ng/g will likely produce milk contaminated with aflatoxin M_1_ levels higher than 0.5 ng/g [14, 15]. However, total aflatoxin concentrations in sunflower seeds and cakes produced in Tanzania have not been analyzed or reported leaving questions such as to what magnitude of the risk to human and animal health from consumption of sunflower products. Therefore, the aim of the present study was to survey total aflatoxin levels in sunflower seed and cake samples collected from multiple micro-scale sunflower oil mills across Tanzania.

## Materials and methods

### Collection of samples

Permission to collect sunflower seeds and cakes from sunflower processors in the following towns of Tanzania was granted by a local authority known as Small Industries Development Organization (SIDO). Neither human nor animal subjects were involved in this survey.

In the year 2014 harvest season (June–July), a total of 86 samples (~200 g each sample) of sunflower seeds (S) and cakes (C) were collected from sunflower processing facilities across Tanzania. The seed samples (S = 42) and seed cake samples (C = 44) were randomly collected from individual sunflower oil extractors in the following towns: Mbeya (S = 7; C = 7), Iringa (S = 7; C = 7), Morogoro (S = 5; C = 5), Dodoma (S = 7; C = 7), Singida (S = 6; C = 6), Babati-Manyara (S = 6; C = 7), and Karatu-Arusha (S = 4; C = 5).

In year 2015 (September–October), a total of 96 samples (~200 g each sample) were collected. The seed samples (S = 48) and seed cake samples (C = 48) were randomly collected from sunflower oil extractors in the following towns: Mbeya (S = 9; C = 9), Iringa (S = 7; C = 7), Morogoro (S = 6; C = 6), Dodoma (S = 7; C = 7), Singida (S = 7; C = 7), Babati-Manyara (S = 7; C = 7) and Karatu-Arusha (S = 5; C = 5).

All samples were placed in polyethylene bags and taken to Sokoine University of Agriculture for aflatoxin analysis. All samples were stored at -20°C prior to analysis.

### Materials and chemicals

Sunflower seeds and cakes were analyzed using the Veratox^®^ for aflatoxin ELISA kit (Cat. No. 8030, Neogen Corporation, Glasgow, UK) consisted of aflatoxin standards 0, 5, 15, 50 ng/g; antibody wells, conjugate, substrate, and stop reagent; Veratox^®^ Mycotoxin Starter Kit (9271A); Mycotoxin Extraction Kit (8052); and Neogen 4700 Micro-well Reader (9303). These materials were purchased from NeogenEurope Corporation (Reg. No. 18634, St Stephen’s House, 279, Bath Street, Glasgow, G2, 4JL, UK). HPLC–grade methanol (Sigma–Aldrich) was used as received. Stock AFB_1_ standard (10 μg in 10 ml methanol) was purchased from (Trilogy Analytical Laboratory Inc. (870 Vossbrink Drive, Washington, MO 63090, USA). De-ionized water was obtained from Sokoine University of Agriculture, Morogoro, Tanzania).

### Aflatoxin analysis

#### Extraction

Aflatoxins from the seed and cakes were extracted using an AOAC-approved method (AOAC-RI 050901) as recommended by Neogen Corporation. A representative sample (~200 g) of seeds or cakes was thoroughly ground into fine powder using a mill grinder (IKA^®^ A11 Basic 07.028450, IKA^®^ Works, Inc., 2635 North Chase, NC 28405–7419, Wilmington, USA). Then, 50 mL of methanol/deionized water (70:30 v/v) were added to the powdered sample (10 g) in a mycotoxin extraction cup (250 mL) to make a suspension, which was vigorously shaken for 3 min. The suspension was allowed to incubate until all particles settled to the bottom. The supernatant solution was then decanted, and filtered into a sample tube using a syringe filled with cotton wool filter. The pH values of all sample solutions ranged from 6.7 to 7.4.

#### Enzyme linked immunosorbent assay

The extracts were assayed for aflatoxins using Veratox^®^ Direct Competitive Enzyme-Linked Immunosorbent Assay (ELISA) in a micro-well format as indicated by the manufacturer (Neogen Corporation, Glasgow, UK) and Manjula and coworkers [16]. The lower limit of detection (LOD) of this assay was 1.4 ng/g. Sample concentrations below the LOD were reported as not detected (n.d.). Sample concentrations that exceeded 50 ng/g (the highest concentration of aflatoxin standard) were further diluted. The diluted sample concentration was multiplied by the dilution factor to obtain the actual concentration of total aflatoxin in the original sample. All samples were analyzed in triplicate to obtain mean concentrations and standard deviations.

#### Recovery of AFB_1_

The sensitivity of the method was determined by calculating percent recovery of aflatoxin for the seeds and cakes. Using a Hamilton-syringe–fixed needle (Lot 719446; Hamilton Company, P.O. Box 10030, Reno, Nevada), aflatoxin-free sunflower seeds and cakes powders (among the samples) (10 g) were spiked with AFB_1_ standard at 10 and 25 ng/g concentrations. AFB_1_-spiked samples were extracted according to AOAC-RI 050901 method to recover AFB_1_. The spiked samples were analyzed in triplicate for each spiked concentration and averages and standard deviations were determined.

#### Statistical analysis

Statistical analysis was performed using SAS software version 9.4 (SAS Institute Inc., Cary, NC). The SAS procedures used were PROC QLIM for multifactorial tobit analysis (allowing for left-censoring at 1.4 ng/g) on aflatoxin concentrations and PROC GLIMMIX for all multifactorial logistic regression analyses of incidence rates, with factors including location, year and their interaction [17]. To render sample concentrations to be more nearly normally distributed for tobit analysis, aflatoxin concentrations were log-log transformed for analyses, with estimates subsequently back-transformed (antilog-antilog) to the scale of observation (i.e, ng/g). Similarly, estimated log odds ratios using logistic regression analyses on incidence rates were back-transformed to incidence rates with approximate standard errors determined using the delta method as provided by the PROC GLIMMIX software. Differences between locations and between years were declared if *P*<0.05.

## Results

### Recovery of AFB_1_

All recoveries of AFB_1_ were greater than 70% (Table 1) indicating the suitability of the approved AFB_1_ extraction protocol (AOAC-RI050901).

**Table 1:** Recovery of AFB_1_ spiked in aflatoxin-free sunflower seed and cakes^a^.

Sample type	Spiked AFB_1_ Concentration (ng/g)	Mean AFB_1_ Recovered (ng/g) ±SD	Recovery (%)
Sunflower seed meal	10	7.1±0.7	71
Sunflower seed meal	25	19.6±1.1	78
Sunflower seed cake	10	8.0±0.6	80
Sunflower seed cake	25	19.4±0.7	78

^a^ Values are means of three determinations; SD = Standard Deviation.

### Concentrations of total aflatoxin in sunflower seeds in the year 2014 harvest season

Table 2 shows the number of contaminated samples, the number of the samples contaminated with concentration above 20 ng/g, mean aflatoxin concentrations and tobit model mean estimates of aflatoxin concentrations in samples from various local oil extractors situated in Babati, Singida, Dodoma, Morogoro, Iringa, Mbeya and Karatu, the major hubs of sunflower processing in Tanzania. Dodoma, Babati, and Singida demonstrated significantly (*P*<0.05) higher mean aflatoxin concentrations (59.6, 46.8, and 45.8 ng/g, respectively) than other towns (Morogoro, 0.7 ng/g; Mbeya, 0.2 ng/g; and Karatu, 1.8 ng/g). Dodoma, Babati, and Singida showed higher ranges (1.7–280.6, 1.8–162.0, and 1.4–261± ng/g, respectively) than Morogoro (1.6–1.9), Mbeya (0–14) and Karatu-Arusha (2.1–2.7 ng/g). Aflatoxins were not detected in samples collected from Iringa. Of seven samples from Dodoma, five were contaminated (71%) and one of the five samples had highest concentration of 280.6 ng/g. Of six samples from Singida, five were contaminated (83%) and one sample contained a maximum concentration of 261.8 ng/g, while 83% of the six samples from Babati were contaminated and the maximum concentration was 162 ng/g. Of seven samples from Mbeya, only one sample (14%) was contaminated (1.4 ng/g). Karatu had only four samples of which three were contaminated and the maximum concentration was 2.7 ng/g.

**Table 2:** Aflatoxin concentrations in sunflower seeds collected from micro- and small-scale sunflower oil processors in Tanzania in the sunflower-harvesting season of 2014.

Location/Town	Number of Samples	Number of Contaminated Samples and Aflatoxin Concentrations (ng/g)†	Number of samples with concentration above 20 ng/g	Mean (ng/g)	Tobit model mean estimate‡ (ng/g)
Babati-Manyara	6	5 [41.3, 1.8, n.d., 3.1, 73.0, 162.0]	3	46.8	4.6^c^
Singida	6	5 [7.7, 1.4, n.d., 261.8, 1.9, 2.0]	1	45.8	2.3^bc^
Dodoma	7	5 [280.6, 32.3, 1.7, 48.9, 54.0, n.d., n.d.]	4	59.6	3.7^c^
Morogoro	5	2 [n.d., 1.6, n.d., n.d., 1.9]	0	0.7	1.2^ab^
Iringa	7	n.d. [n.d., n.d., n.d., n.d., n.d., n.d., n.d.]	0	n.d.	1.0
Mbeya	7	1 [n.d., n.d., 1.4, n.d., n.d., n.d., n.d.]	0	0.2	1.1^a^
Karatu-Arusha	4	3 [2.1, 2.7, 2.4, n.d.]	0	1.8	1.8^ab^

Notes

† n.d. = not detected (n.d.<LOD); LOD = limit of detection (1.4 ng/g).

‡Any two estimates not sharing the same letter indicate means are different from each other (*P<*0.05). The Tobit estimates are based on a Tobit regression analysis. It essentially is a standard censored regression analysis that allows for the fact that all responses below the LOD (1.4) ARE NOT all really equal to 0. In fact, it is quite likely that all the responses below the LOD are really non-zero. The Tobit regression analysis respects the fact that responses are **left-censored** below 1.4 (i.e., all one knows is that a n.d. is below 1. But that is still useful information for a Tobit analysis. The Tobit estimates are based on a projected mean response below the LOD; if the sample sizes were much larger, we’d have even better estimates. One might notice that there were some n.d. in other locations that also had detectable responses; all of the data information (those above and below the LOD) are used to provide the Tobit estimates for those estimates as well.

### Concentrations of total aflatoxin in sunflower seed cake in the year 2014 harvest season

Sunflower cakes were collected from the same locations indicated in Table 2. The aflatoxin concentrations, mean aflatoxin concentrations, and tobit model mean estimates of aflatoxin concentrations in sunflower cake samples are indicated in Table 3. It should be noted that cake samples collected and reported in Table 3 were residues of seeds that were different from the seed samples reported in Table 2. Sunflower cakes from Dodoma, and Mbeya had higher average aflatoxin concentrations (33.5, and 28.8 ng/g, respectively) than the other locations (Singida, 13.2; Morogoro, 10.8; Babati, 6; Iringa, 2.9; and Karatu, 1.5 ng/g). The concentrations ranged from 1.9 to 88.2 and 2.8 to 97.7 ng/g in Dodoma and Mbeya, respectively. Lower ranges of aflatoxin concentrations were observed in Singida (2.0–34.3), Morogoro (2.2–31.9), Babati (1.7–17.8), Iringa (1.7–5.3) and Karatu (1.5–2.2 ng/g). The highest aflatoxin concentration (98 ng/g) was observed in one of the 6 samples collected from Mbeya. One out of seven samples from Dodoma was highly contaminated (88.2 ng/g). All cake samples from Dodoma, Singida, Babati and Iringa were contaminated, whereas sample contamination frequencies from Morogoro, Mbeya and Karatu, were 80, 86 and 80%, respectively. Locations in which nearly all samples were contaminated but with aflatoxin concentrations below the action level of 20 ng/g were Karatu (2.2 ng/g), Iringa (5.3 ng/g) and Babati (17.8 ng/g). The maximal aflatoxin levels in Singida and Morogoro samples were 34.3 and 31.9 ng/g, respectively.

**Table 3:** Aflatoxin concentrations in sunflower seed cakes collected from micro- and small-scale sunflower oil processors in Tanzania in the sunflower-harvesting season of 2014.

Location/Town	Number of Samples	Number of Contaminated Samples and Aflatoxin Concentrations (ng/g): †	Number of samples with concentration above 20 ng/g	Mean (ng/g)	Tobit model mean estimate (ng/g) ‡
Babati-Manyara	7	7 [1.7, 1.9, 2.1, 3.3, 17.8, 6.0, 9.0]	0	6.0	3.3^ab^
Singida	6	6 [17.9, 10.6, 10.3, 34.3, 4.2, 2.0]	1	13.2	7.1^b^
Dodoma	7	7 [45.3, 46.3, 2.4, 46.8, 3.8, 88.2, 1.9]	4	33.5	8.3^b^
Morogoro	5	4 [3.5, 16.2, 31.9, 2.2, n.d.]	1	10.8	3.2^b^
Iringa	7	7 [2.6, 5.3, 1.8, 1.7, 3.7, 3.3, 2.1]	0	2.9	2.5^ab^
Mbeya	7	6 [2.8, n.d., 87.2, 7.8, 97.7, 3.0, 3.2]	2	28.8	4.2^b^
Karatu-Arusha	5	4 [2.2, 1.5, n.d., 1.7, 2.2]	0	1.5	1.6^a^

Notes

† n.d. = not detected (n.d.<LOD); LOD = limit of detection (1.4 ng/g).

‡ Any two estimates not sharing the same letter indicate means are different from each other (*P<*0.05). The Tobit estimates are based on a Tobit regression analysis. It essentially is a standard censored regression analysis that allows for the fact that all responses below the LOD (1.4) ARE NOT all really equal to 0. In fact, it is quite likely that all the responses below the LOD are really non-zero. The Tobit regression analysis respects the fact that responses are **left-censored** below 1.4 (i.e., all one knows is that a n.d. is below 1. But that is still useful information for a Tobit analysis. The Tobit estimates are based on a projected mean response below the LOD; if the sample sizes were much larger, we’d have even better estimates. One might notice that there were some n.d. in other locations that also had detectable responses; all of the data information (those above and below the LOD) are used to provide the Tobit estimates for those estimates as well.

### Concentrations of total aflatoxin in sunflower seed samples in the year 2015 harvest season

Table 4 shows aflatoxin concentrations, mean aflatoxin concentrations, and tobit model mean estimates of aflatoxin concentrations in sunflower seed samples collected in the sunflower-growing season of year 2015. Samples from Morogoro, Singida, and Mbeya showed significantly (*P<*0.05) higher average concentrations (118.6 ng/g, 33.8 ng/g, and 21.1 ng/g, respectively) than Iringa (5.7 ng/g), Karatu (1.6 ng/g), Babati (1.2 ng/g) and Dodoma (0.5 ng/g). Fifty percent (3/6) of the samples from Morogoro were contaminated and one sample (16.7%) showed a very high concentration (663 ng/g). Eighty-six percent (6/7) of the samples from Singida, were contaminated with concentrations ranging from 1.6–217.6 ng/g. Eighty-nine percent (8/9) of the samples from Mbeya were contaminated and the highest concentration was 174 ng/g (range: 1.4–174.2 ng/g). Eighty-six percent (6/7) samples from Iringa were contaminated (range: 1.5–28.6 ng/g). Dodoma had the lowest range (1.6–20 ng/g) and 29% (2/7) of the samples were contaminated. Sixty-percent (3/6) of the samples from Karatu were contaminated (range: 1.9–3.7 ng/g).

**Table 4:** Aflatoxin concentrations in sunflower seeds collected from micro- and small-scale sunflower oil processors in Tanzania in the sunflower-harvesting season of 2015.

Location/Town	Number of Samples	Number of Contaminated Samples and Aflatoxin Concentrations (ng/g)†	Number of samples with concentration above 20 ng/g	Mean (ng/g)	Tobit model mean estimate (ng/g) ‡
Babati-Manyara	7	4 [n.d., n.d., 2.3, n.d., 1.4, 1.9, 1.4]	0	1.2	1.3^ab^
Singida	7	6 [10.7, 1.6, n.d., 1.8, 217.6, 2.3, 2.6]	1	33.8	2.5^b^
Dodoma	7	2 [n.d., n.d., n.d., n.d., n.d., 1.6, 2.0]	0	0.5	1.2^a^
Morogoro	6	3 [46.3, 2.8, n.d., 662.7, n.d., n.d.]	2	118.6	2.1^b^
Iringa	7	6 [2.4, 28.6, n.d., 1.5, 3.6, 2.6, 1.5]	1	5.7	2.1^b^
Mbeya	9	8 [n.d., 2.5, 174.2, 1.4, 2.3, 1.9, 2.0, 3.3, 1.9]	1	21.1	2.1^ab^
Karatu-Arusha	5	3 [n.d., n.d., 1.9, 3.7, 2.3]	0	1.6	1.6^ab^

Notes

† n.d. = not detected (n.d.<LOD); LOD = limit of detection (1.4 ng/g).

‡ Any two estimates not sharing the same letter indicate means are different from each other (*P<*0.05). The Tobit estimates are based on a Tobit regression analysis. It essentially is a standard censored regression analysis that allows for the fact that all responses below the LOD (1.4) ARE NOT all really equal to 0. In fact, it is quite likely that all the responses below the LOD are really non-zero. The Tobit regression analysis respects the fact that responses are **left-censored** below 1.4 (i.e., all one knows is that a n.d. is below 1. But that is still useful information for a Tobit analysis. The Tobit estimates are based on a projected mean response below the LOD; if the sample sizes were much larger, we’d have even better estimates. One might notice that there were some n.d. in other locations that also had detectable responses; all of the data information (those above and below the LOD) are used to provide the Tobit estimates for those estimates as well.

### Concentrations of total aflatoxin in sunflower seed cake samples in year 2015 harvest season

The aflatoxin concentrations, mean aflatoxin concentrations, tobit model mean estimates of aflatoxin concentrations in sunflower seed cakes are listed in Table 5. As noted above, cake samples collected, analyzed and reported in Table 5 were residues of seeds that were different from the seed samples reported in Table 4. Morogoro and Dodoma samples had higher average aflatoxin concentrations of 149.0 and 120.6 ng/g, respectively as compared to Singida (11.3 ng/g), Mbeya (7.1 ng/g), Babati (3.7 ng/g), Iringa (2.2 ng/g), and Karatu (1.8 ng/g). Based on their ranges, Dodoma (13.3–598.4 ng/g), Morogoro (2.7–536 ng/g), Mbeya (1.4–20.3 ng/g), and Singida (3.2–52.8 ng/g) were the only locations that showed maximum concentrations in their cakes above the action level of 20 ng/g. Iringa, Karatu, and Babati had lower ranges of 1.5–12, 1.7–11.2, and 1.5–13.8 ng/g in cakes, respectively; while all of the samples from Morogoro were contaminated with very high aflatoxin concentrations with one having a maximum concentration of 536.0 ng/g. Fifty-seven percent of the samples from Dodoma were contaminated with a maximum concentration of 598.4 ng/g.

**Table 5:** Aflatoxin concentrations in sunflower seed cakes collected from micro- and small-scale sunflower oil processors in Tanzania in the sunflower-harvesting season of 2015.

Location (Town)	Number of Samples	Number of Contaminated Samples and Aflatoxin Concentrations (ng/g) †	Number of samples with concentration above 20 ng/g	Mean (ng/g)	Tobit model mean estimate (ng/g) ‡
Babati-Manyara	7	5 [n.d., 13.8, 1.5, 2.1, n.d., 1.5, 6.8]	0	3.7	1.8^ab^
Singida	7	4 [n.d., 15.7, 3.2, n.d., 52.8, n.d., 7.1]	1	11.3	2.2^abc^
Dodoma	7	4 [111.0, 121.2, n.d., n.d., n.d., 598.4, 13.3]	3	120.6	3.4^c^
Morogoro	6	6 [229.4, 40.9, 2.7, 536.0, 10.1, 74.7]	4	149.0	26.7^d^
Iringa	7	3 [n.d., 12.0, n.d., n.d., n.d., 1.5, 1.9]	0	2.2	1.4^a^
Mbeya	9	9 [1.4, 7.5, 5.0, 4.9, 3.2, 1.5, 3.2, 17.1, 20.3]	0	7.1	3.5^b^
Karatu-Arusha	5	2 [n.d., 1.7, n.d., n.d., 11.2]	0	1.8	1.4^ab^

Notes

† n.d. = not detected (n.d.<LOD); LOD = limit of detection (1.4 ng/g).

‡ Any two estimates not sharing the same letter indicate means are different from each other (*P<*0.05).). The Tobit estimates are based on a Tobit regression analysis. It essentially is a standard censored regression analysis that allows for the fact that all responses below the LOD (1.4) ARE NOT all really equal to 0. In fact, it is quite likely that all the responses below the LOD are really non-zero. The Tobit regression analysis respects the fact that responses are **left-censored** below 1.4 (i.e., all one knows is that a n.d. is below 1. But that is still useful information for a Tobit analysis. The Tobit estimates are based on a projected mean response below the LOD; if the sample sizes were much larger, we’d have even better estimates. One might notice that there were some n.d. in other locations that also had detectable responses; all of the data information (those above and below the LOD) are used to provide the Tobit estimates for those estimates as well. These cake samples collected and reported in Table 5 are residues of seeds that were different from the seed samples reported in Table 4.

### Statistical analysis

Our statistical analysis indicated no evidence of interaction in aflatoxin incidence rates in cakes between year and location. However, there was a strong overall location effect across the two years. Interestingly, there was a significant interaction between locations and years for aflatoxin incidences in sunflower seeds. The statistical data showing the incidence rates of aflatoxin contamination in location and across the two years are shown in Table 6.

**Table 6:** Incidence rates of aflatoxin contamination for each location across and within the two years of 2014 and 2015.

Factor	Overall incidence rates of aflatoxin contamination beyond LOD across the two years (incidence rate ±standard deviation) (ng/g) †		Year-specific incidence rates of aflatoxin contamination beyond LOD in sunflower seeds and cakes (incidence rate ± standard deviation) (ng/g) †			
			Year 2014		Year 2015	
	Seeds	Cakes	Seeds	Cakes	Seeds	Cakes
Location						
Babati-Manyara	0.65±0.13^ab^	0.90±0.08^ab^	0.71±0.17^a^	1.00±0.00^a^	0.57±0.19^ab^	0.71±0.17^a^
Singida	0.85±0.10 ^b^	0.83±0.11^ab^	0.83±0.15^a^	1.00±0.00^a^	0.86±0.13 ^ab^	0.57±0.19^a^
Dodoma	0.50±0.14 ^ab^	0.83±0.10^ab^	0.71±0.17^a^	1.00±0.00^a^	0.29±0.17^a^	0.57±0.19^a^
Morogoro	0.44±0.15 ^ab^	0.94±0.06^ab^	0.40±0.22^a^	0.80±0.18^a^	0.50±0.20 ^ab^	1.00±0.00^a^
Iringa	0.43±0.14 ^a^	0.76±0.12^ab^	0.00±0.00^a^	1.00±0.00^a^	0.86±0.13 ^ab^	0.43±0.19^a^
Mbeya	0.55±0.13 ^ab^	0.96±0.04^b^	0.14±0.13^a^	0.86±0.13^a^	0.89±0.11^b^	1.00±0.00^a^
Karatu-Arusha	0.60±0.16 ^ab^	0.63±0.17^a^	0.60±0.22^a^	0.80±0.18^a^	0.60±0.22 ^ab^	0.40±0.22^a^
Year						
2014	0.48±0.08^a^	0.95±0.03^b^				
2015	0.68±0.07 ^a^	0.70±0.07^a^				

† Estimates not sharing the same letter within the same factor (i.e. location or year) and within the same column are different from each other. LOD = Limit of detection (1.4 ng/g).

## Discussion

Sunflower is an important oilseed crop in Tanzania that comprises about 36% of the total cooking oil consumed in the country each year [9]. Besides cooking oil, sunflower seeds are a source of sunflower cakes used for dairy and beef cattle and poultry feedstuffs. Tanzanians also consume roasted and raw sunflower seeds as a food snack. There are a few reports in the literature indicating that *A*. *flavus* and *A*. *parasiticus* can infect sunflower and cause aflatoxin accumulation in seeds and cakes [18, 19, 20, 21]. However, there are no reports on potential aflatoxin contamination of sunflower seeds and cakes grown in Tanzania. The available aflatoxin reports focus mainly on aflatoxin contamination of agricultural commodities such as maize, cassava, and market-cured fish [16, 22, 23]. The present study aimed at determining aflatoxin levels in sunflower seed and cake samples collected from micro- and small-scale sunflower oil processors in various locations within Tanzania.

To investigate the extent and recurring nature of aflatoxin contamination in sunflower seeds and cakes, two surveys were carried out across the country in two consecutive years. Whereas the overall aflatoxin incidence rate in cakes was greater in 2014 than 2015 (Table 6), there was no any significant difference in overall aflatoxin incidence rate in seeds between the two years (*P*>0.05). The aflatoxin concentrations presented in Tables 2, 3, 4 and 5 clearly demonstrate that many sunflower seed and cake samples collected in 2014 and 2015 were contaminated particularly those from Singida, Dodoma, Morogoro and Babati, which whose many samples were contaminated with high aflatoxin concentrations above the regulatory limit of 20 ng/g. Singida and Dodoma are in lowland central Tanzania where local climate patterns are characterized by semi-arid, warm and drought conditions, which are favorable environments for growth of aflatoxigenic molds [24]. Although there is not a semi-arid climate in Morogoro, high temperature, high humidity, and drought characterize this location and make it favorable for aflatoxigenic mold colonization. The climatic features of these locations shown in the supplementary local meteorological data (S1 Fig) indicate that between January and May (the sunflower cultivation months) of 2014, Singida and Dodoma had received less rainfall than the rest of the study locations, suggesting drought conditions. Drought causes stress to plants in the field, thus rendering them more susceptible to fungal infestation and insect damage, and exacerbating aflatoxin accumulation in seeds [25, 26]. Moreover, harsh climatic conditions influence the distribution, density, and the structure of *A*. *flavus* and *A*. *parasiticus* communities and susceptibility of crop plants to such communities [24, 27, 28]. Thus, perhaps these harsh conditions may have orchestrated the aflatoxin contamination of the sunflower seeds in the field and carried over to the storage facilities before processing and to cakes after processing.

In contrast, lower aflatoxin incidence rates occurred in regions with moderate climatic conditions, higher altitude, and adequate rainfall; growth of aflatoxin-producing fungi are less favored in such conditions. These considerations may explain why the aflatoxin means estimates in highland locations such as Iringa, Mbeya, and Karatu were relatively lower (Tables 2, 3, 4 and 5 and S1 Fig) as compared to Singida, Manyara, Dodoma and Morogoro. However, poor storage facilities and poor post-harvest handling of seeds and cakes are additional risk factors for increased aflatoxin accumulation in stored crops and can offset the advantages of moderate climatic conditions [25, 29]. For example, despite its moderate temperature, Mbeya had high mean estimate of aflatoxin concentration in cakes collected in both years and in seeds collected in 2015 (Tables 3, 4 and 5). Thus, the findings for Mbeya, Morogoro, Dodoma and Singida could be due to a collection of risk factors, which are probably poor storage facilities, post-harvest handling and drought.

Inadequate rainfall received during the sunflower growing season of 2014 between March and April (S1 Fig), the critical period when sunflower flowering and seed maturation usually occurs, might have caused sunflower plants and seeds to become susceptible to fungal infections, colonization and consequent aflatoxin contamination in the field. The harvested seeds entering the storage facilities from the field may also have microflora and insects, which can introduce respiratory activity and raise moisture content of the seeds. Consequently, molds carried over with the seeds from the field to the storage facilities may have continued to grow and accumulate aflatoxin in the seeds prior to processing. Although Morogoro is characterized by high temperature and relative humidity (S1A Fig and S1C Fig), the adequate rainfall (S1B Fig) it received in 2014 could have alleviated the severity of fungal contamination and aflatoxin levels. If Morogoro had received less rainfall in 2014, perhaps the extent of aflatoxin contamination would have been similar to that of Dodoma since these regions are contiguous. This assumption was true for Morogoro findings of 2015 (Tables 4 and 5) because this location overall received less rainfall in 2015 than 2014.

The preliminary findings in 2014 prompted us to carry out another survey in 2015 to investigate the recurrence of aflatoxin contamination in sunflower seeds and cakes in the same locations where samples were collected in 2014 (Tables 4 and 5). Overall, there was significantly (*P*<0.05) more aflatoxin contamination of sunflower cakes in 2015 than in 2014, but there were no significant differences in the overall incidence rates of aflatoxin contamination in sunflower seeds between the two years. However, there were significant differences in aflatoxin incidence rates in seeds and cakes between locations in each year. These differences between locations exist because climatic conditions and post-harvest practices vary from location to location. In addition, sanitation and ventilation conditions of the storage facilities vary from one processing oil mill to another within one location, and from one location to another.

When we compare the aflatoxin incidence rates in seeds and cakes collected from various locations in 2015 to those collected from the same locations in 2014 (Tables 2, 3, 4 and 5), we observe that the mean estimates of aflatoxin concentration for locations of Singida, Dodoma, Morogoro and Mbeya appear repetitively and significantly higher than the rest of the locations in both years. However, there is considerable inconsistency which may have been caused by variability in sanitation practices among processing plants within a location and among locations in the two years. The small number of samples is probably another contributing factor for such inconsistency. Also, it is possible that these high mean estimates of aflatoxin concentrations above 20 ng/g, in large part could be attributed to drought caused by semi-arid climate in Singida, and Dodoma, and high temperatures and humidity in Morogoro [24, 29, 30, 31]. This attribution cannot hold true for Mbeya because this location has moderate temperature, humidity and fairly adequate rainfall. Perhaps other risk factors such as poor post-harvest storage of the seeds and cakes could account for its higher mean estimates across the two years.

Storage duration could be another important factor that might account for higher aflatoxin contamination in cakes in year 2015 than 2014. While the samples collected in year 2014 had stayed in storage for 3 months (May–July 2014) prior to sample collection, a subset of samples collected in 2015 had been in storage for 5 months (May–September 2015), which suggests that the longer the storage period of seeds or cakes, the higher incidence rates of aflatoxin contamination becomes. This factor is likely enhanced by poor storage facilities and conditions.

The current problem of climate change may increase the risk of aflatoxin contamination in susceptible crops in the near future [24, 27, 28, 32]. *A*. *flavus* and *A*. *parasiticus* distribution is predicted to increase with global warming [33]. Drought, high humidity and high temperature cause stress to plants and reduce production of phytoalexins, the chemicals used by plants to resist fungal infection [34]. When such conditions prolong, crop plants in the field weaken and are rendered susceptible to fungal infection [24, 35]. It has been reported that Dodoma and a large part of Central Tanzania is often characterized by drought due to inadequate, unpredictable rainfall and increase in temperature [31]. The semi-arid climatic conditions in Central Tanzania (Dodoma, and Singida) might have contributed to the observed high levels in such locations because these conditions are conducive to aflatoxigenic molds to thrive. Deforestation, wild fires and charcoal production on Uluguru mountains in Morogoro have resulted in gradual micro-climate change manifested by lower precipitation and higher temperatures [36]. This may likely expand the ecological environment of aflatoxigenic molds in Morogoro as well.

Like seeds, cakes are vulnerable to aflatoxin contamination. In the two surveys, 80% of 92 sunflower cake samples were contaminated with aflatoxin ranging from 1.4 to 598.4 ng/g, compared to only 59% of 90 sunflower seed samples contaminated with aflatoxin ranging from 1.4 to 662.7 ng/g illustrating that generally more cake samples were contaminated than seeds. This may be because aflatoxin contamination is concentrated in a small number of seeds, which may have not been sampled; but when the highly-contaminated seeds are ground into cakes, aflatoxin becomes detectable. This could be considered as a “concentration effect” of seed pressing. For example, aflatoxin could not be detected in sunflower seed samples from Iringa in 2014, but was detected in its sunflower cake samples (mean = 2.9 ng/g and range = 1.7–5.3 ng/g) although in low levels (Tables 2 and 3). Additionally, since the cakes and seeds are stored in the same storage warehouse, there is likely cross-contamination of spores from the seeds and the floor of storage facility to the cakes, which may be stored longer than the seeds.

These surveys were not without a challenge. It was hard to obtain samples from sacks situated deep in the interior of sack piles in the stores [37]. We obtained samples only from the sacks peripherally located in each store. The small sample size per region was also a limitation. Larger sample numbers would provide more accurate information on the incidence and extent of aflatoxin contamination, and provide a better measure of variability in aflatoxin levels from one region to another. We therefore recommend that future survey studies should address these limitations. However, despite these limitations, the results still indicated significant incidence rates of aflatoxin contamination in sunflower seeds and cakes particularly from locations of central Tanzania.

The results for seven locations of Tanzania (Tables 2, 3, 4 and 5) corroborate reports from Sudan [6], Pakistan [19], Spain [20], Iran [21], and India [38, 39], which indicated that sunflower seeds and cakes were susceptible to aflatoxin contamination. The high aflatoxin levels in samples obtained from Dodoma, Morogoro, Babati and Singida towns may have resulted from a combination of drought and sub-optimal postharvest handling and storage of seeds and cakes [24, 31, 36]. Anecdotal information from the sunflower oil processors (buyers of the sunflower seeds) revealed that unscrupulous sellers might adulterate sound seeds with moldy seeds to increase the weight of the packed sunflower seed bags for economic profit. Unfortunately, smaller quantities of moldy seeds in the midst of sound seeds may serve as reservoirs for increasing aflatoxin contamination in storage.

Climatically, the eastern and central provinces of Kenya and the central regions of Tanzania may have similar semi-arid conditions, which are favorable for the S-strain of *A*. *flavus* implicated with the eastern and central Kenya aflatoxicosis outbreak in 2004 [40, 41]. This wild strain is known for its ability to produce high quantities of aflatoxins and it is more prevalent in such stressed environments [40, 41]. However, prevalence of this fungal strain in central Tanzania is not known and it is a potential gap for future study.

To summarize: In our study, 59% of 90 sunflower seed samples and 80% of 92 cake samples collected from sunflower oil processors across Tanzania were contaminated with total aflatoxins. Moreover, 14% of seed samples and 17% of cake samples were contaminated with total aflatoxin concentrations above the action level of 20 ng/g, with several samples having in the several hundred ng/g of aflatoxin. These high aflatoxin levels in a commodity frequently consumed by the Tanzanian population indicate that local authorities must implement interventions to prevent and control aflatoxin contamination along the sunflower commodity value chain, to enhance food and feed safety in Tanzania. Follow-up research is needed to determine intake rates of sunflower seed products in humans and animals, to inform exposure assessments and to better understand the role of sunflower seeds and cakes as a dietary aflatoxin source.

## Supporting information

S1 Fig **Meteorological data showing maximum monthly mean temperature (°C)–(A); total monthly rainfall (mm)-(B); monthly mean relative humidity (%)–(C); and region altitude (meters)–(D).** Source: Tanzania Meteorological Authority (TMA), Ubungo Plaza, 3rd Floor, P. O. Box 3056, Dar es Salaam, United Republic of Tanzania. Data were obtained in 2015.
(PDF)

S1 Dataset Aflatoxin levels in sunflower cake and seed samples collected from individual mills located in seven regions of Tanzania in 2014 and 2015. (XLSX)
