# Supplementary material for: Aflatoxin levels in sunflower seeds and cakes collected from micro- and small-scale sunflower oil processors in Tanzania
Source: PLoS One. 2017 Apr 18;12(4):e0175801. doi: 10.1371/journal.pone.0175801 (PMC5395219; doi:10.1371/journal.pone.0175801)
Supplement: S1 Fig — Meteorological data showing maximum monthly mean temperature (°C)–(A); total monthly rainfall (mm)-(B); monthly mean relative humidity (%)–(C); and region altitude (meters)–(D). Source: Tanzania Meteorological Authority (TMA), Ubungo Plaza, 3rd Floor, P. O. Box 3056, Dar es Salaam, United Republic of Tanzania. Data were obtained in 2015. (PDF) [file pone.0175801.s001.pdf]

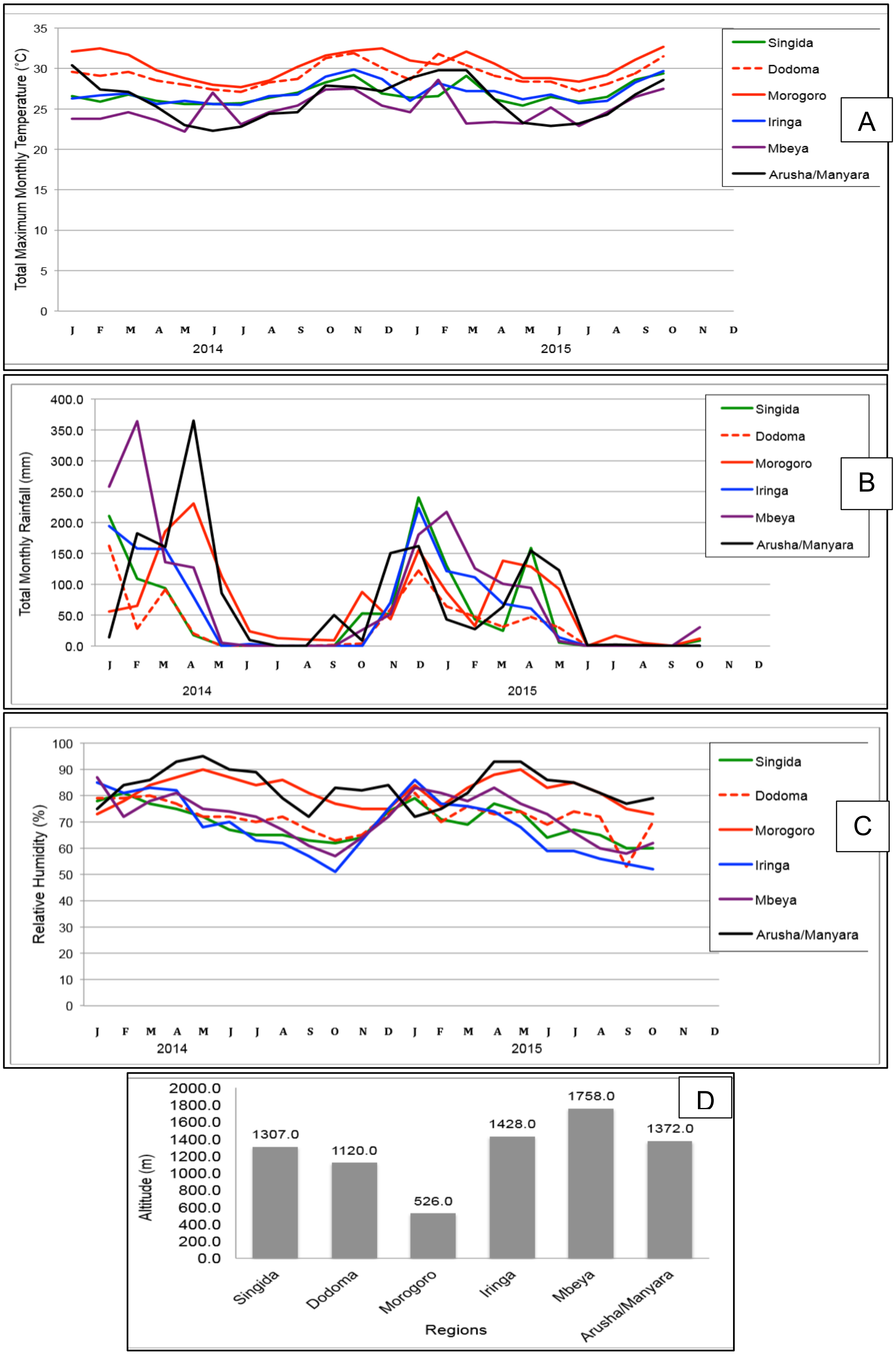


**S1 Fig. Meteorological data showing maximum monthly mean temperature (˚C)–(A); total monthly rainfall (mm)-(B); monthly mean relative humidity (%)–(C); and region altitude (meters) –(D).** Source: Tanzania Meteorological Authority (TMA), Ubungo Plaza, 3rd Floor, P. O. Box 3056, Dar es Salaam, United Republic of Tanzania. They were obtained in 2015.
